# Naringenin-Loaded Solid Lipid Nanoparticles: Physical–Chemical Characterization and In Vitro Antibacterial Activity

**DOI:** 10.3390/ph18020232

**Published:** 2025-02-08

**Authors:** Federica De Gaetano, Francesco Caridi, Noemi Totaro, Consuelo Celesti, Valentina Venuti, Giovanna Ginestra, Antonia Nostro, Silvana Tommasini, Cinzia Anna Ventura, Rosanna Stancanelli

**Affiliations:** 1 Department of Chemical, Biological, Pharmaceutical and Environmental Sciences, University of Messina, V.le Ferdinando Stagno D’Alcontres 31, 98166 Messina, Italy; noemi.totaro@studenti.unime.it (N.T.); giovanna.ginestra@unime.it (G.G.); antonia.nostro@unime.it (A.N.); stommasini@unime.it (S.T.); rstancanelli@unime.it (R.S.); 2 Department of Mathematical and Computer Sciences, Physical Sciences and Earth Sciences, University of Messina, V.le Ferdinando Stagno D’Alcontres 31, 98166 Messina, Italy; fcaridi@unime.it (F.C.); vvenuti@unime.it (V.V.); 3 Department of Engineering, University of Messina, Contrada Di Dio, 98166 Messina, Italy; ccelesti@unime.it

**Keywords:** naringenin, solid lipid nanoparticles, technological characterization, physical–chemical characterization, in vitro antibacterial activity, biofilm inhibition

## Abstract

Currently, problems related to antibiotic resistance are shifting the focus of pharmaceutical research towards natural molecules with antibacterial properties. Among them, flavonoids represent promising molecules with strong antibacterial features; however, they have poor biopharmaceutical properties. In this study, we developed solid lipid nanoparticles (SLNs) loaded with the flavanone naringenin (NRG) to offer an option for treating bacterial infections. NRG-SLNs systems were prepared by a solvent emulsification/diffusion and ultrasonication method, using Compritol^®^ 888 ATO (COM) as the lipid. The optimal formulation was obtained using a 10% (*w*/*w*) theoretical amount of NRG (NRG_10_-SLNs), exhibiting homogeneous sizes (approximately 50 nm and 0.15 polydispersity index), negative zeta potential (−30 mV), and excellent encapsulation parameters (an encapsulation efficiency percentage of 97.9% and a drug content of 4%). NRG_10_-SLNs presented good physical stability over 4 weeks. A cumulative drug release of 55% in 24 h and the prolonged release of the remaining amount over 10 days was observed. In addition, µ-Raman spectroscopy, differential scanning calorimetry, thermogravimetric analysis, and X-ray diffraction measurements were carried out to characterize the drug–lipid interactions. Finally, the in vitro antibacterial and antibiofilm activities of NRG_10_-SLNs were assayed and compared to free NRG. NRG_10_-SLNs were bacteriostatic against *Staphylococcus aureus,* including the methicillin-resistant *S. aureus* (MRSA) and *Escherichia coli* strains. An improvement in the antibacterial activity of NRG-loaded SLNs compared to the free molecule was observed against *S. aureus* strains, probably due to the interaction of the surfactant-coated SLNs with the bacterial surface. A similar trend was observed for the biofilm inhibition.

## 1. Introduction

Over the years, the increasing use of antibiotics in medicine has given rise to the ever-growing problem of antimicrobial resistance, which is equally alarming during both hospital and non-hospital treatment. Consequently, there is an increasing need to develop new therapeutic strategies with the ability to circumvent bacterial resistance. In this context, natural antimicrobial agents are considered a promising approach in counteracting the spread of difficult-to-treat microorganisms [1]. Among them, flavonoids are a group of bioactive molecules derived from plants, with their antibacterial activity being widely recognized in the literature [2,3]. Due to their natural origins, flavonoids attain a higher consumer compliance than chemically synthesized drugs. Moreover, they have an advantage over antibiotics due to their multiple actions on cells, meaning that it is less likely that bacteria will develop resistance to them [4]. Specifically, studies suggest that the antibacterial activity of flavonoids occurs through different mechanisms: (i) inhibition of nucleic acid synthesis, (ii) inhibition of cytoplasmic membrane function, and (iii) inhibition of energy metabolism [2]. Furthermore, flavonoids are relatively non-toxic, low cost, and readily available.

Naringenin (5,7,4′-trihydroxy flavanone; NRG) (Figure S1) is an aglycone belonging to a group of flavanones that have demonstrated interesting pharmacological effects on biological systems, including anti-inflammatory, anticancer, and antioxidant activity [5]. NRG also shows interesting antimicrobial activity against a variety of bacteria [6], such as *Escherichia coli*, *Streptococcus mutans,* and pathogenic multidrug-resistant bacteria such as methicillin-resistant *Staphylococcus aureus* (MRSA) [7,8]. The activity of NRG is stronger against Gram-positive than Gram-negative bacteria, as demonstrated by Wang et al. [9] in a comparative study of NRG activity against *S. aureus* and *E. coli*. NRG caused a dose-dependent growth rate inhibition, which was higher in *S. aureus* than in *E. coli.* It appears that low concentrations of NRG produced variations in the membrane protein conformation and fatty acid composition, disrupting the *S. aureus* biofilm, whilst at high concentrations, NRG significantly improved membrane fluidity in the bacteria [9]. Furthermore, NRG can bind to *S. aureus* DNA and can inhibit quorum sensing, thus limiting biofilm formation [7,9]. NRG could modify the signaling mediated by AI-2 (population-sensing signaling molecule), produced by intestinal flora and involved in the communication between various bacterial species [10]. NRG is also active against *S. mutans,* inducing the total inhibition of biofilm formation at a dose of 200 μg/mL. The study demonstrated that NRG is able to improve the surface hydrophobicity of the bacteria and decrease aggregation [11]. Another interesting NRG activity is the inhibition of the α-toxin produced by different *S. aureus* strains, which was evaluated by measuring the reduction in hemolytic activity with respect to the control. Zhang et al. demonstrated that, in mice infected with the *S. aureus* strain that causes pneumonia, a significant reduction in the inflammation area was achieved after treatment with 100 μg/Kg of NRG, when compared to the non-treated animals [12]. This effect was probably due to a reduction in pro-inflammatory cytokines, as demonstrated in a study involving pediatric patients with bronchial pneumonia. The authors demonstrated that treatment with NRG decreased IL-6, IL-8, and TNF-α levels, whilst increasing the amount of anti-inflammatory IL-10 [13].

It is evident that NRG implements different mechanisms against Gram-positive bacteria, thus its development as an antibacterial drug could represent a key factor in overcoming antibiotic resistance. Unfortunately, NRG possesses low water solubility (4.38 μg/mL) [14], intestinal instability, and extensive first-pass metabolism, which negatively affects its oral bioavailability (only 15% of ingested NRG is absorbed in the human gastrointestinal tract) [15], limiting its clinical application. New formulative strategies are needed to overcome these drawbacks, improving solubility, stability, and bioavailability, and permitting the controlled release of NRG [16] in the treatment of various pathologies. Complexation or conjugation with cyclodextrins (CDs) represents an excellent strategy to improve the water solubility of both synthetic drugs [17,18] and natural compounds [19,20,21,22,23]. For example, native and modified β-CD improved NRG hydrophilicity [24], particularly when complexation was combined with a controlled pH and the presence of polysorbate [25]. Furthermore, the complexation increased NRG permeation through the Caco-2 monolayer and improved its oral bioavailability (approximately 7-fold and 15-fold for AUC and Cmax, respectively), representing a suitable option for the development of NRG as a therapeutic agent in the treatment of dyslipidemia, diabetes, and HCV infection [26]. Polymeric nanoparticles also possess interesting properties in delivering both synthetic [27,28,29] and natural compounds [30,31,32]. Polymeric NRG nanoformulations with increased NRG bioavailability and effectiveness in different pathologies have been developed. Hybrid Chitosan/β-CD nanoparticles potentiated the antibacterial and antibiofilm activities of NRG, achieving ~34% growth inhibition and ~60% biofilm inhibition towards the biosensor *E. coli* Top 10 strain [33]. NRG-loaded PLGA nanoparticles for nose-to-brain delivery were able to prevent neurodegeneration induced by Paraquat in an in vivo model of Parkinson’s disease by regulating oxidative stress markers [34], thus representing a valid alternative to synthetic drugs in Parkinson’s disorder treatment. The neuroprotective effects of NRG-loaded chitosan nanoparticles were demonstrated in vitro in the SH-SY5Y cell line [35]. Within the large panorama of nanocarriers, excellent results in improving the therapeutic performance of natural products were obtained by using lipid- based nanocarriers such as liposomes, microemulsions, solid lipid nanocarriers (SLNs), and nanostructured lipid carriers (NLCs) [36,37]. Among them, SLNs represent the most performant carrier as an alternative to traditional colloidal carriers [38,39,40,41]. SLNs are fabricated using a variety of biodegradable lipids and lipidic stabilizers without the use of any organic solvent, are generally recognized as safe (GRAS), and whose sizes, polydispersity, surface charge, short- and long-term stability, drug loading, and release profile can be modulated using a selection of lipids and surfactants. They can be prepared for a wide variety of administration routes, offering several advantages compared to the other nano-scale systems, i.e., the ability to incorporate lipophilic or hydrophilic drugs, targeting options, controlled release, good bioavailability and stability, good tolerability, and a low risk for acute and chronic toxicity [42,43].

Studies are available in the literature describing NRG-SLNs development, although the majority are aimed at the mere physical–chemical characterization of the system or an evaluation of the antioxidant, anti-inflammatory, and anticancer efficacy of the nanoformulation. For example, SLNs are able to enhance the cytotoxic effects of NRG against pancreatic cancer cell lines [44] or to improve the neuroprotective effects of NRG in the rotenone-induced Parkinson’s disease rodent model, showing the potential to avert the progression of the disease [45]. Pre-treatment of PC12 cell cultures with NRG-SLNs resulted in higher cell survival, increased mitochondrial membrane potential, and reduced autophagic marker expression with respect to free NRG, demonstrating NRG-SLNs formulation as a promising candidate to protect cells from streptozocin-induced neurotoxicity [34]. After intragastric administration in rats, the oral bioavailability of NRG-SLNs was approximately 2.93-fold higher than that of NRG solution, demonstrating SLNs as a safe and effective oral delivery system to enhance the therapeutic potential of NRG in rheumatoid arthritis treatment [46]. A 2.53-fold increase in bioavailability with respect to NRG suspension was also observed as a consequence of the pulmonary administration of NRG-SLNs nanoformulations, showing good potentiality for SLNs as NRG carriers [47].

To the best of our knowledge, no published papers have evaluated the effectiveness of SLNs formulations in improving the antimicrobial activity of NRG. There is a panoramic lack in the research pertaining to new antibacterial agents; however, due to the interesting antimicrobial properties shown by NRG, this could be largely improved by advancing SLNs nanotechnology. For this reason, in this work we developed NRG-SLNs formulations, and assayed their antibacterial effectiveness in vitro against *Staphylococcus aureus,* including methicillin resistant *S. aureus* (MRSA) as the Gram-positive bacteria, and *Escherichia coli* and *Pseudomonas aeruginosa* as the Gram-negative bacteria. Furthermore, the effect on biofilm formation in the *S. aureus* strains was evaluated.

## 2. Results and Discussion

### 2.1. Technological Characterization of NRG-SLNs

The lipophilic nature of NRG prevented its pharmaceutical formulation in liquid and solid dosage forms. Thus, to overcome its solubility issue and obtain a sustained release, we formulated SLNs in which the bioactive molecule was entrapped in the matrix core.

NRG_x_-SLNs were prepared using different NRG amounts, ranging from 1 to 10%, by means of the solvent emulsification/diffusion method. The diffusion of organic solvents containing lipids and NRG into the aqueous solution facilitated the formation of colloidal suspensions that then underwent ultrasonication to ensure the formation of nanosized systems. COM was used as a lipid phase. This is a mixture of glycerol behenate that presents low cytotoxicity and, due to its amphiphilic properties [48], the ability to efficiently entrap hydrophilic and lipophilic drugs, thus controlling their release [49]. As reported by other authors [47], the amount of lipid and surfactant used in the preparation process influences the physical–chemical properties of the SLNs. The lipid and surfactant composition of the NRG-SLNs and the operative conditions were established experimentally in a previous study [41]. The amount of Tween 80 used as a surfactant was chosen based on the sizes of the final formulations. The correct amount permits the generation of SLNs with small sizes; however, an excessive amount could produce foam. Concerning the solvent, we selected ethanol because NRG demonstrates good solubility in this solvent at room temperature, while COM is soluble at temperatures higher than 60 °C. This difference permits separation of the lipid from the NRG, permitting an indirect evaluation of the NRG encapsulated in the SLNs. The COM melts at a temperature higher than 75 °C, thus the emulsification was carried out at 80 °C; the successive cooling down at room temperature permitted the solidification of the lipids into a spherical matrix, coated by Tween 80 and entrapping NRG [47]. On the other hand, the amount of resulting lipid and surfactant is a determinant for establishing the optimal properties of all lipid-based formulations for NRG delivery, such as simple [50] or hybrid nanoemulsions [51].

The encapsulation parameters for NRG_x_-SLNs are reported in Table 1. For all the investigated samples, a high E.E. % and D.C. % and a good Yield % were achieved. The formulation prepared with the highest NRG theoretical amount (10%) resulted in the highest performance as far as E.E. % (97.9%, *w*/*w*) and D.C. % (4.1%, *w*/*w*) were concerned. The obtained E.E.% demonstrated a very high affinity of NRG for COM and no loss of the drug. Similar results were obtained by Sahu et al. [52] using COM as a lipid, but different surfactants (poloxamer 188 and soya lecithin) and in higher amounts than the Tween 80 present in our nanoformulation. On the other hand, other authors reported lower E.E.% for NRG-SLNs with respect to our nanoparticles, but using glycerylmonooleate as a lipid [47,53], confirming the high affinity of NRG for COM.

Due to the optimal E.E. % and D.C. % values for the NRG_10_-SLNs formulation, this was chosen in order to proceed with the successive characterizations.

#### 2.1.1. Particle Sizes, Polydispersity Index, and Zeta Potential

Table 2 shows the sizes, polydispersity index (PDI), and zeta potential (ζ) values of empty SLNs and NRG_10_-SLNs samples, before and after lyophilization. As far as hydrodynamic diameters (D_H_) are concerned, the very small size of SLNs was obtained following an optimized preparation of empty SLNs and NRG_10_-SLNs using a precise quantity of Tween^®^ 80 (0.5% *w*/*v*), according to the methods reported in previous works [39,54,55]. Furthermore, PDI (%) values were determined to be ~0.15, indicating that there were no clusters present and that the population of SLNs was homogeneous in the investigated samples. Finally, both empty SLNs and NRG_10_-SLNs exhibited high negative ζ values, suggesting that high electrostatic repulsive forces exist between neighboring particles that inhibit aggregation and ensure high physical stability in the systems. No variation in these parameters was observed after lyophilization.

To confirm the physical stability of NRG_10_-SLNs over time, we maintained the nanoformulation for 30 days in different conditions of light and temperature, then measured the mean sizes, PDI, and ζ values. No appreciable changes were observed in that time (Table 3), demonstrating the good physical stability of the system.

#### 2.1.2. In Vitro Drug Release Studies

**Figure 1:**
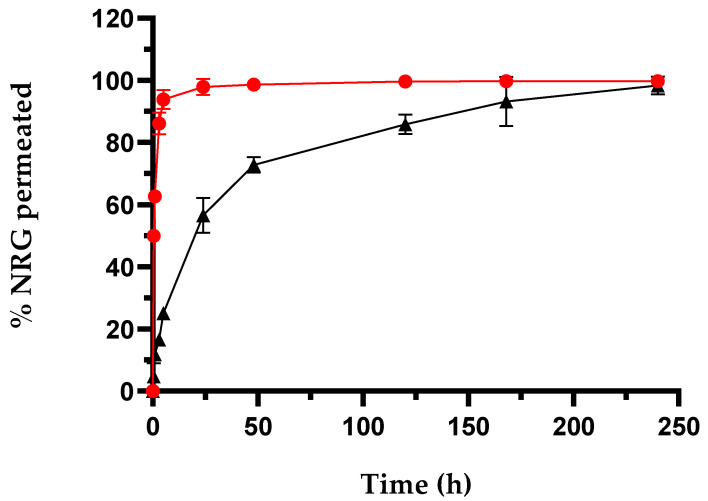
In vitro release profiles of NRG_10_-SLNs (black squares), comparative to free NRG (red circles). The results are expressed as mean values from three different experiments and three different batches ± S.D. If bars are not visible, they are within the symbol. All values for the NRG_10_-SLNs (excluding 0, 168, and 240 h) are statistically significant compared to the free RTN data (*p* < 0.001).

NRG release from SLNs was performed using the dialysis method by suspending a weighted amount of NRG_10_-SLNs in a PBS:EtOH mixture (85:15, *v*/*v*) and comparing to a solution of free NRG in the same solvent (Figure 1). The quantitative appearance of free NRG was observed in the dialysis medium after approximately 4 h. This trend demonstrated the suitability of the dialysis bag for these studies and allowed us to hypothesize that the release of NRG, when encapsulated in SLNs, is controlled by the SLNs themselves.

NRG_10_-SLNs exhibited a release profile in which about 25% of the encapsulated NRG was quickly released within the first 5 h of the experiment, followed by a sustained release that saw a cumulative amount of NRG approximating 55% within 24 h, and the remaining 45% of encapsulated NRG slowly released within 10 days. The NRG released in the first 5 h is probably located on or near the surface of the SLNs. The remaining amount is probably uniformly distributed throughout the lipidic matrix and takes a longer time to complete the release. Faster release of NRG was obtained by other authors from SLNs produced using COM or other lipids. For example, SLNs produced with glycerilmonoleate released NRG quantitatively within 60 h, even if the SLNs possess larger sizes (>200 nm) with respect to our nanoformulation [53]. This difference may be due to the excellent affinity of NRG for COM, and the resulting drug–lipid association. In order to migrate through the lipid matrix, NRG–lipid dissociation must occur, thus contributing to the resulting delay in the diffusion process [56]. On the other hand, it is not only the type of lipid used that determines the release rate of NRG. In fact, Sahu et al. [52], synthesized NRG-SLNs using COM with poloxamer 188 and soya lecithin as the surfactant and co-surfactant. They detected a release of NRG within 24 h, demonstrating that the type of surfactant also plays an important role in drug release in this system.

Data obtained from the release studies were treated using three different kinetic models, i.e., zero-order, first-order, and the Higuchi model, together with the Korsmayer-Peppas transport model [57,58,59]. In order to identify the most suitable among the kinetic models, the linear regression coefficients (R^2^) of all models were compared. As evidenced by Table 4, the highest R^2^ value (~1), indicative of a good fit [57], was obtained for the first-order kinetic model.

According to the literature [60,61] it is well-established that, depending on the type of lipid and the morphology of the SLNs, the release mechanisms involved can be of different types, i.e., diffusion of the drug, swelling, or degradation of the lipid matrix. Specifically, in the case of the NRG_10_-SLNs system and given the lipophilic nature of the matrix, the swelling mechanism is excluded. Furthermore, considering that the in vitro release studies were conducted in the absence of enzymes, lipid degradation can be ruled out. Thus, diffusion represents the unique mechanism underlying NRG release. Based on the Korsmeyer-Peppas model, the value of the transport exponent (n) allows us to recognize the drug release mechanism. The obtained n value ~0.58 indicates a non-Fickian diffusion as the predominant release mechanism for NRG by the NRG_10_-SLNs system [59].

### 2.2. Chemical–Physical Characterization of NRG_10_-SLNs

**Figure 2:**
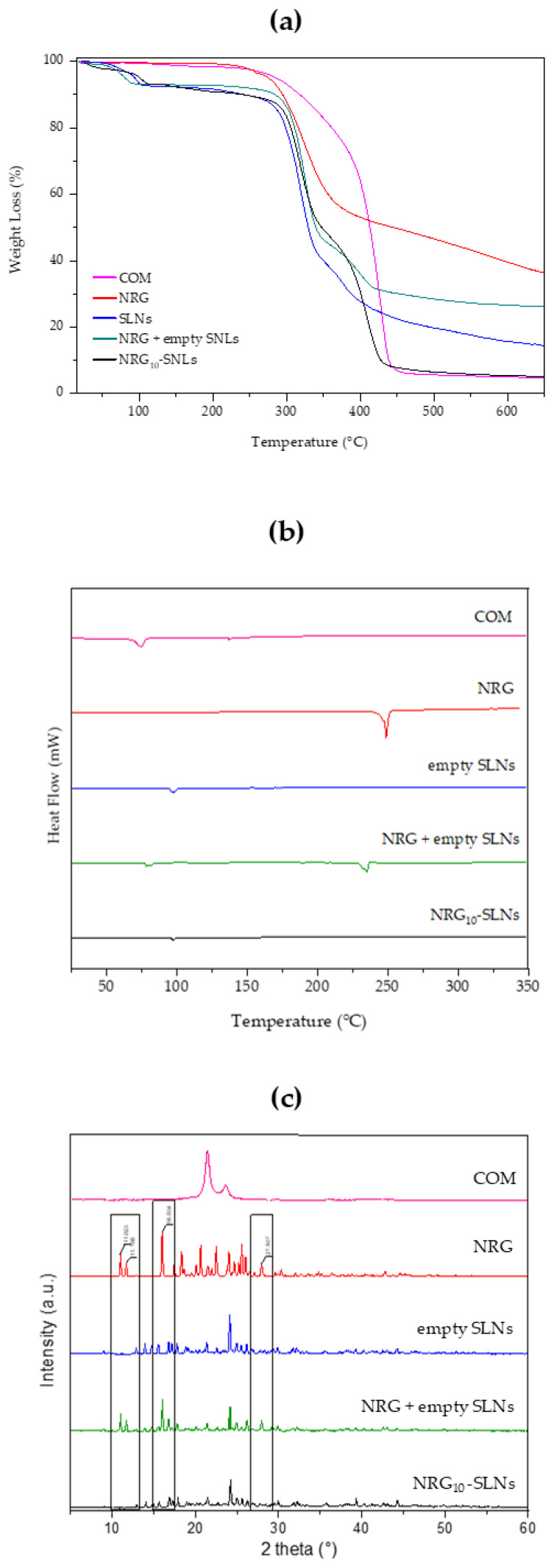
TGA thermograms (**a**), DSC analysis (**b**), and X-ray diffraction patterns (**c**) of COM (pink line), NRG (red line), empty SLNs (blue line), NRG+SLNs physical mixture (green line), and NRG_10_-SLNs (black line).

TGA analyses were performed in an inert environment at a heating rate of 10 °C/min. For each sample, the TGA analysis was evaluated up to 700 °C to investigate the thermal stability of free NRG and NRG_10_-SLNs. Figure 2a shows the TGA profiles of COM, free NRG, empty SLNs, the NRG_10_-SLNs system, and an NRG + empty SLNs physical mixture. Firstly, the ~7% weight loss observed for empty SLNs, the NRG_10_-SLNs nanoformulation, and the physical mixture in the temperature range 55–110 °C, is due to the loss of water absorbed during the preparation process. On the other hand, the same thermal event is absent from the profile of COM, which shows a 2% loss of weight at higher temperatures (from 120 °C to 250 °C). For all samples, we observed the onset of degradation at a temperature up to 280 °C. Particularly, from derivative of the TG curve (Figure S2 in Supplementary Materials) we can observe the degradation of free NRG at 335 °C, corresponding to the maximum slope on the TG curve (see red line in Figure 2a). The NRG_10_-SLNs pyrolysis curves showed an increased stability in NRG as a consequence of its encapsulation, evidenced by the derivative of the TG curve (see black line in Figure S2) as an intense signal of degradation at about 425 °C, almost overlapping with the degradation of empty SLNs. The presence of a weak signal on the NRG_10_-SLNs derivative curve at about 280 °C could reflect the degradation of the low amount of free NRG present on the surface of the SLNs nanoformulation. The physical mixture, on the other hand, undergoes gradual degradation starting at a lower temperature with respect to the nanoformulation (see green line in Figure S2), probably reflecting the presence of NRG as a non-encapsulated molecule.

The freeze-dried NRG_10_-SLNs sample was subjected to a calorimetric analysis to determine the state of the incorporated molecules. In Figure 2b, DSC curves of COM, free NRG, empty SLNs, the physical mixture of empty SLNs and NRG, and the NRG_10_-SLNs formulation are shown. Due to the chemical composition of COM (it is composed of non-pure triacylglycerols), its DSC curve shows two endothermic peaks, the main one at around 75 °C, probably due to metastable polymorphic forms [48], and the second one at approximately 136 °C. A shift towards higher temperatures was observed for the first peak, and the disappearance of the peak at 136 °C could be as a consequence of the thermal stress suffered by the lipid during SLNs preparation (see empty SLNs). NRG shows an endothermic melting peak at 250 °C, indicative of the crystalline state of the molecule. The physical mixture exhibits two endothermic peaks: one at 240 °C due to the presence of free NRG and the other at about 90 °C corresponding to the SLNs. However, in the DSC curve of the NRG_10_-SLNs, the endothermic peak for the drug disappeared, revealing that NRG interacts with the lipid, via hydrogen bonds or van Deer Waals interactions, and is mostly located inside the SLNs.

The X-ray diffraction study was employed to ascertain the state of NRG after encapsulation in the system. The XRD spectra of COM, free NRG, empty SLNs, the physical mixture of empty SLNs and NRG, and NRG_10_-SLNs are shown in Figure 2c. Free NRG and the physical mixture present crystalline properties with very sharp peaks in the 2 theta range from 3° to 40°, while COM showed a single diffraction peak at 2 theta ~21.0°. Sharp peaks were observed in the diffractograms of the empty SLNs, demonstrating a different crystal structure for the lipids in the matrix compared to the bulk solid lipids. The XRD spectra of the physical mixture show clear peaks of NRG (indicated by black rectangles), evidencing the presence of NRG in a crystalline state. However, the disappearance of the main free NRG peaks in the diffractograms for the NRG_10_-SLNs could be due to the incorporation of NRG into the lipid crystal lattice [41] and the creation of non-covalent NRG-SLNs interactions. This drug–lipid association could be responsible for the sustained release of NRG previously observed. Dissociation of this complex is needed to permit the migration of NRG through the lipid matrix, producing as a consequence a retardation in the diffusion process [56].

**Figure 3:**
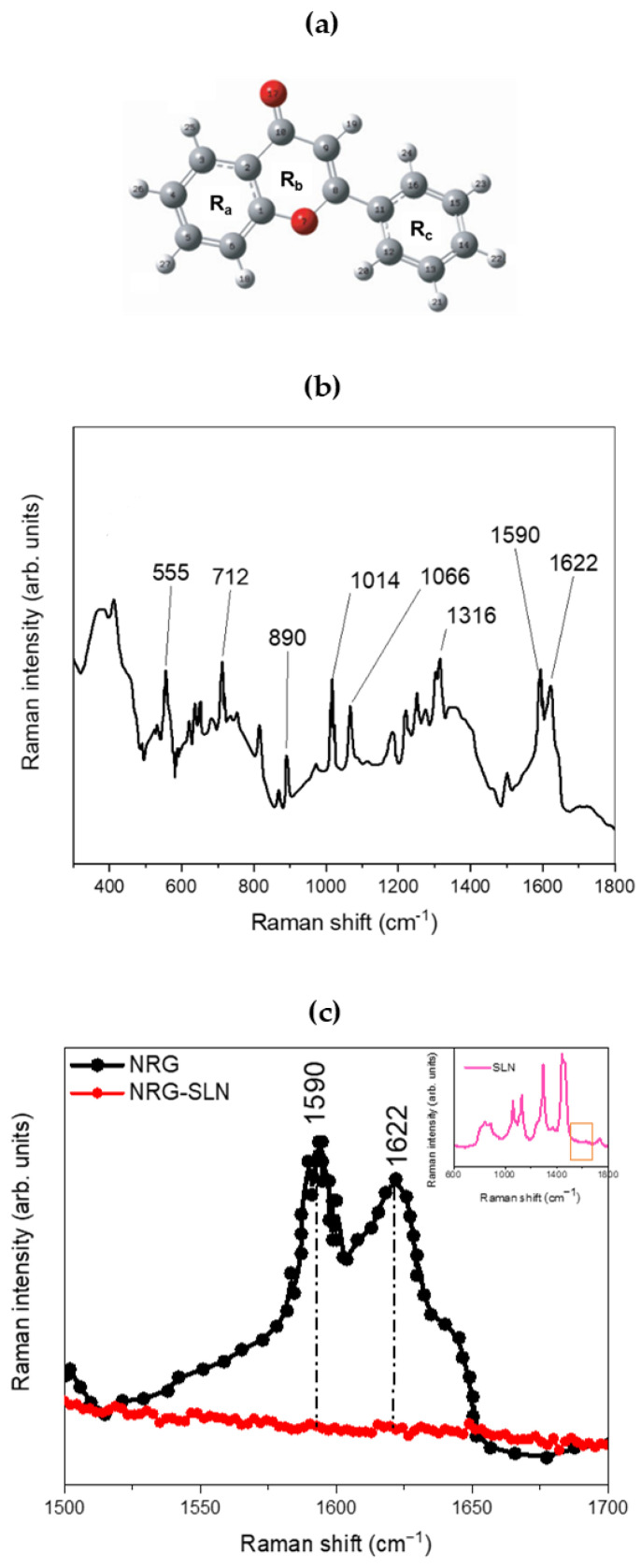
(**a**) Structure and atomic numbering of NRG. In particular, the positions of the carbon atoms are represented in gray, and those of the oxygen atoms are represented in red. (**b**) The experimental µ-Raman spectrum of NRG. (**c**) Experimental µ-Raman spectra, in the 1500–1700 cm^−^^1^ wavenumber range, respectively associated with NRG (black line) and NRG_10_-SLNs (red line). In the inset, the experimental µ-Raman spectrum of empty SLN is reported (pink line), evidencing the 1500–1700 cm^−1^ wavenumber range, free of vibrational contributions from the solid–lipid matrix.

Figure 3 shows the structure (panel a) and the experimental µ-Raman spectrum of NRG (panel b and c). The latter, although characterized by an extremely composite profile, shows a series of main bands that extend within a wavenumber range from ~500 cm^−1^ to ~1800 cm^−^^1^. The peak at ~555 cm^−^^1^ may be associated with OCC bending of the R_a_ and R_b_ rings. The peak at ~712 cm^−^^1^ can be associated with the O=CCC out-of-plane bending mode, while the contribution centered at ~890 cm^−^^1^ is due to the CCO bending modes, both attributable to the R_b_ ring. Again, the pair of contributions at ~1014 cm^−^^1^ and ~1066 cm^−^^1^, clearly visible within the investigated range, can be attributed, respectively, to HCCC torsions involving the R_c_ ring and to the CO stretching modes of the R_a_ ring, while the contribution at ~1316 cm^−^^1^ is attributable to the C-OH (C_5_–OH) deformation modes of the R_b_ ring. Within the 1500–1700 cm^−^^1^ spectral range, it is possible to observe a broadband constituted by the envelope of two distinct vibrational contributions, centered at ~1590 cm^−^^1^ and ~1622 cm^−^^1^, and attributable, respectively, to the CC stretching of the R_a_, R_b,_ and R_c_ rings and to the CO (C_10_=O_17_) stretching modes of the R_b_ ring [62,63]. Finally, contributions deriving from the CH stretching modes associated with the R_a_ and R_c_ rings are also present in the wavenumber range from ~2900 cm^−1^ to ~3100 cm^−1^. Figure 3c shows the experimental µ-Raman spectra, collected in the 1500–1700 cm^−1^ wavenumber range, respectively associated with NRG and the NRG_10_-SLNs system. The focusing of attention on this spectral region is mainly due to the latter being free of vibrational contributions from the solid–lipid matrix (see inset of Figure 3c), thus allowing us to directly observe variations in the vibrational bands of the NRG as a consequence of the drug entrapment within the investigated matrix.

From the comparison between the µ-Raman spectra associated with NRG and NRG_10_-SLNs, we observe a clear reduction in the intensity of the vibrational modes centered at ~1590 cm^−^^1^ and ~1622 cm^−^^1^ and attributable, as previously described, to the CC stretching modes of the R_a_, R_b_, and R_c_ rings (~1590 cm^−^^1^), and to the CO (C_10_=O_17_) stretching modes of the R_b_ ring (~1622 cm^−^^1^) of NRG. This reduction, as already justified in previous reports on similar systems [30], suggests a *hindering* of the functional groups responsible for the aforementioned vibrations, as a consequence of the activation of *“host–guest”* interactions that drive the encapsulation of the drug in the studied SLNs.

**Figure 4:**
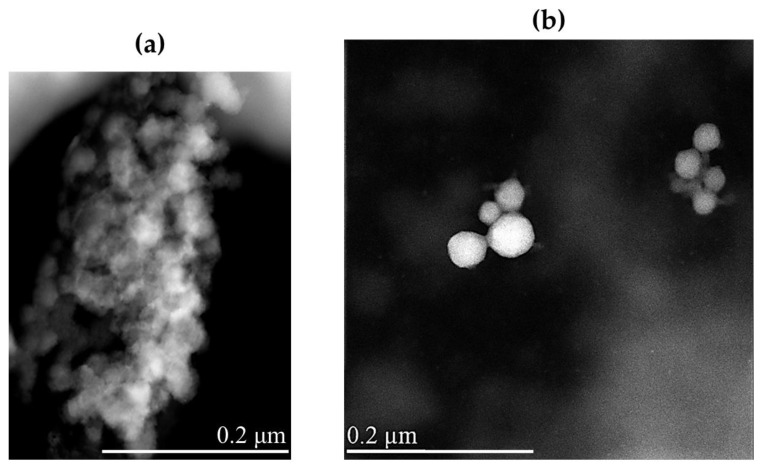
STEM images of lyophilized NRG_10_-SLNs before (**a**) and after (**b**) redispersion. The bar width in the panels indicates the size.

Morphological characterization of lyophilized NRG_10_-SLNs was carried out, before and after redispersion, by scanning transmission electron microscopy (STEM) (Figure 4). The solid sample appears as an aggregate powder, probably due to the presence of trehalose, used as a cryoprotectant, that wraps around the nanoparticles and keeps them close to each other (Figure 4a). The redispersed powder showed spherical nanoparticles with a smooth surface and a very low degree of aggregation (Figure 4b).

### 2.3. Antibacterial Activity

The results of the antibacterial tests are shown in Table 5. NRG possesses bacteriostatic activity rather than bactericidal effects, showing an MIC value of 0.5 mg/mL against *S. aureus* ATCC 6538 and *S. aureus* (MRSA) ATCC 43300 and 1 mg/mL against *E. coli.* No activity was evident against *P. aeruginosa* at the highest concentration assayed. Similar results were obtained by Wang et al. [9] against *S. aureus* and *E. coli*, evidencing that the latter was less susceptible to NRG treatment. The cell wall structure in Gram-positive and Gram-negative bacteria and the mechanism of action of NRG may be responsible for the difference in activity [64]. Gram-negative bacteria possess a complex outer membrane that is less permeable and provides an additional selective barrier that can effectively hinder the access of hydrophobic compounds such as NRG to the cytoplasmic membrane [9].

Regarding the nanoformulation, empty SLNs showed antibacterial activity with MIC values of 1 and 0.5 mg/mL toward *S. aureus* ATCC 6538 and *S. aureus* (MRSA) ATCC 43300, respectively. On the contrary, no activity was reported against either Gram-negative bacteria (*E. coli* and *P. aeruginosa*) at the highest concentration assayed. It is conceivable to suppose that the surfactant (Tween 80^®^) used for the nanoparticles preparation and adsorbed on their surface could be responsible for the antibacterial effect. Indeed, Tween 80 is known to interact with the bacterial surface, enhancing cell permeability and membrane fluidity [65,66]. Specifically, Tween 80 at concentrations of 0.25% and 0.5% has been reported to reduce the viability of planktonic *S. aureus* cells and the biofilm formation capacity [67]. Furthermore, Maurya et al. [65], demonstrated that Tween 80 adsorbed onto SLNs efficiently disrupts *S. aureus* biofilms.

NRG_10_-SLNs showed, in terms of MIC values, similar (against *S. aureus* ATCC 6538) or higher (against *S. aureus* ATCC 43300) antibacterial activity, with respect to free NRG, and always higher with respect to empty SLNs (see Table 5). It can be assumed that the permeabilizing activity exerted on the cell membrane by Tween 80, which coats the SLNs, facilitates the entry of NRG released by the nanoformulation into the bacterial cell, where it directly targets nucleic acids, thus enhancing its action. Furthermore, some considerations need to be made taking into account the release profile of NRG from SLNs. 

**Figure 5:**
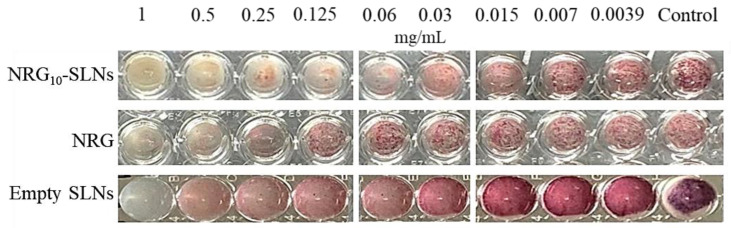
Inhibition of the metabolic activity of *S. aureus* ATCC 6538 cells after the addition of triphenyl tetrazolium chloride (TTC) 0.125% (*w*/*v*).

The nanoformulation progressively releases NRG (see Figure 1), reaching a cumulative amount of 55% in 24 h, i.e., an amount of NRG available to interact with the bacteria equal to about half of the dose of NRG when free in the medium. Despite this, the antibacterial activity of the NRG loaded-SLNs was similar or improved with respect to NRG alone, highlighting the role of the nanocarrier in strengthening the activity of the molecule. In addition, the NRG_10_-SLNs inhibited bacterial metabolic activity more effectively, resulting in a lower presence of viable cells up to 0.06 mg/mL, compared to free NRG (Figure 5). This is probably due to the progressive release of NRG from the SLNs allowing its continuous availability and distribution to the bacterial cells. These observations suggest that, for a combination of different reasons, SLNs are effective carriers for NRG.

**Figure 6:**
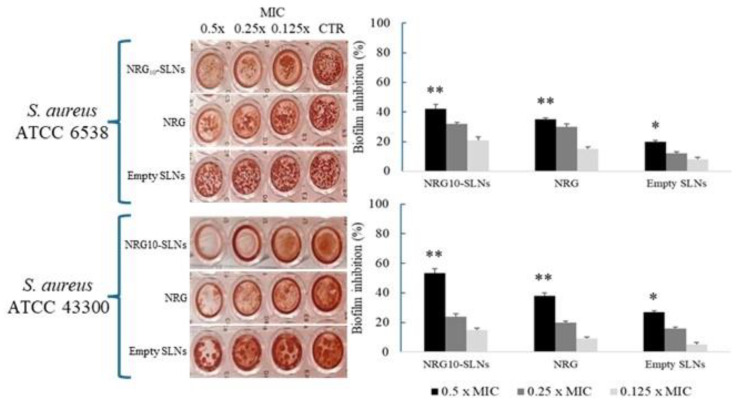
Effects of different concentrations (from 0.5× MIC to 0.125× MIC) of NRG10-SLNs, free NRG, and empty SLNs on biofilm formation in *S. aureus* ATCC 6538 and *S. aureus* ATCC 43300 (MRSA). Data are presented as mean ± SD, * *p* < 0.05, ** *p* < 0.01 vs. control.

Based on the antibacterial activity observed against the two *S. aureus* strains, we evaluated the influence of sub-MIC concentrations of NRG_10_-SLNs, free NRG, and empty SLNs on biofilm formation (Figure 6). The results show the highest inhibition of biofilm production for NRG_10_-SLNs with respect to the free NRG. Notably, a biofilm reduction of 42% and 53% with respect to the free NRG (reduction of 35% and 38%) for *S. aureus* ATCC 6538 and *S. aureus* ATCC 43300 MRSA, respectively, was detected.

The activity of NRG on microbial biofilm is acknowledged in the literature. In this regard, Wen et al. [68] reported the inhibiting effect of NRG on *S. aureus* adhesion to solid surfaces and the subsequent formation of biofilm, due to a reduction in bacterial surface hydrophobicity and esopolysaccharide production. Furthermore, it is also known that interference with biofilm formation may be due to the physical action of the surfactant on the substrate surface [69]. These observations could explain the improvement in the antibiofilm activity of NRG_10_-SLNs with respect to the free NRG.

## 3. Materials and Methods

### 3.1. Materials

Naringenin (MW 272.26, NRG ≥ 95%) was purchased from Aldrich Chemical Company, Inc (Milwaukee, WI, USA); Compritol^®^ 888 ATO (COM) was kindly supplied by Gattefossé SAS (Lyon, France); Tween^®^ 80 and trehalose from Saccharomyces Cerevisiae ≥ 99% were purchased from Sigma Aldrich (St. Louis, MO, USA). Ethanol (EtOH) HPLC grade is a Merck^®^ (Darmstadt, Germany) product. Double-distilled water filtered through 0.22 µm Millipore^®^ GSWP filters (Bedford, MA, USA) was used throughout the study. All other products and reagents were of analytical grade. Dialysis bags were Spectra/Por^®^ 18 × 11.5 mm Dialyse-Membrane (MWCO: 1000, Spectrum Laboratories, Inc., Rancho Dominguez, CA, USA).

### 3.2. Preparation of Empty SLNs and NRG-SLNs

Empty SLNs were prepared using the method reported by Butani et al. [70], with slight modifications. Briefly, 50 mg of COM was solubilized in 2.5 mL of EtOH to form the lipid phase, whereas the aqueous phase was prepared by adding Tween^®^ 80 (0.5% *w*/*v*) to 11.25 mL of double-distilled water [71]. Initially, the two phases were heated at a temperature above 80 °C to ensure the complete melting of the lipid and total dissolution of the surfactant. The Ultra-Turrax T 25 (IKA-Werke, Staufen, Germany) was used during the addition of the lipid phase into the aqueous phase (within 7 min) to ensure high-speed homogenization (11,000 rpm). In order to obtain a particle size as small as possible, the resulting colloidal dispersions were immersed in an ultrasound bath (Bandelin Sonorex^®^ RK 514, amplitude delivered ranges between 3 and 5 µm, Berlin, Germany) for 5 min. After that, the colloidal dispersions were allowed to cool down to room temperature by placing them on a magnetic stirrer (500 rpm) for 24 h to ensure complete removal of the organic solvent. The obtained sample underwent centrifugation at 5000 rpm for 20 min (Heraeus Megafuge 16, ThermoFisher Scientific, Waltham, MA, USA) to remove the COM that did not interact. The pellet was removed and the supernatant containing the SLNs was collected, trehalose (5%, *w*/*v*) was added as a cryoprotectant, and the solution was lyophilized (−53 °C, 43 mTorr) for 72 h (BenchTop K Series Freeze Dryers, VirTis Gardiner, Gardiner, NY, USA).

The SLNs loaded with NRG (NRG-SLNs) were prepared using the same procedure described above, by solubilizing NRG in the heated ethanolic solution of COM at different concentrations, from 1 to 10% (*w*/*w*), based on the weight of lipid (namely, NRG_x_-SLNs, with x = 1, … 10). After centrifugation of the obtained colloidal suspension at 5000 rpm, the supernatant containing NRG_x_-SLNs was added to trehalose (5%, *w*/*v*) and lyophilized for 72 h. The pellet, containing the non-encapsulated NRG and the non-interacting lipid, was used for the indirect determination of encapsulated NRG. Specifically, the pellet was added to cold EtOH (5 mL) and magnetically stirred at room temperature for 5 min to solubilize the NRG. The non-interacting COM remained in suspension since it is insoluble in ethanol at temperatures below 60 °C. Thus, the suspension was filtered through 0.22 µm PTFE filters (Millipore^®^), and the NRG in the filtrate was quantified by UV–Vis spectroscopy as described in Section 3.5 [72]. To ensure the full recovery of NRG, this process was repeated until no bioactive molecule was detected. The encapsulated NRG was determined as the difference between the total amount of NRG added during the preparation of SLNs and the non-encapsulated NRG present in the pellet. The experiment was performed in triplicate and the results were expressed as mean ± standard deviation (S.D.).

### 3.3. Technological Characterization of NRG-SLNs

To determine the yield percentage (Yield %), the lyophilized empty SLNs and the NRG_x_-SLNs were weighed, and the following formula (Equation (1)) was used:

(1) $$Yield\%=\frac{Effective\;weight}{Theoretical\;weight}\times 100$$

The entrapment efficiency percentage (E.E. %) and the drug content percentage (D.C. %) were determined using the following equations (Equations (2) and (3)):

(2) $$E.E.\%=\frac{NRG\;initially\;added-free\;NRG\;outside\;SLNs}{NRG\;initially\;added}\times 100$$

(3) $$D.C.\%=\frac{NRG\;initially\;added-free\;NRG\;outside\;SLNs}{recovered\;SLNs}\times 100$$

Mean particle sizes, polydispersity index (PDI), and zeta potential (ζ) of empty SLNs and NRG_10_-SLNs were measured before and after lyophilization using a Zeta sizer 3000 instrument (Malvern Panalytical Ltd., Malvern, UK) with a Dynamic Light Scattering (DLS) technique. Before the analysis, the samples were diluted 100 times using water stored at a specific temperature to prevent multiple scattering effects. The measurements were repeated three times for accuracy.

### 3.4. In Vitro Drug Release Studies

The lyophilized NRG_10_-SLNs containing 1 mg of NRG were suspended in 5 mL of phosphate buffer solution (PBS, pH 7.4). The suspension was transferred into a pre-activated dialysis bag and then placed in a beaker containing 150 mL of PBS:EtOH mixture (85:15, *v*/*v*). EtOH was added to avoid the precipitation of NRG (water solubility of ~4.38 μg/mL) [73,74]. Throughout the experiment, the dialysis medium was constantly agitated with a magnetic stirrer at 300 rpm and maintained at 37.0 ± 0.5 °C (Telesystem 15.40 thermostat bath with Telemodul 40 C control unit). To ensure sink conditions, the medium was periodically replaced with a preheated (37 °C) fresh mixture of PBS:EtOH (85:15, *v*/*v*). All collected volumes were evaporated under vacuum at 25.0 ± 0.1 °C, and the resulting residues were dissolved in 2 mL of EtOH. The samples were then analyzed by UV–Vis spectroscopy [72], as described in Section 3.5.

Free NRG (1 mg), corresponding to the same amount contained in the NRG_10_-SLNs, was solubilized in a PBS–EtOH mixture (85:15, *v*/*v*) and subjected to dialysis to verify the suitability of the chosen bag. The same procedure performed for NRG_10_-SLNs was followed and NRG was determined by UV–Vis spectroscopy (see Section 3.5).

All experiments were performed in triplicate and data were expressed as the mean value ± S.D. The acquired data for NRG_10_-SLNs were analyzed using the Korsmayer-Peppas transport model in conjunction with three distinct kinetic models, namely the zero-order, first-order, and Higuchi models [57,58,59].

### 3.5. UV–Vis Apparatus and Method Validation

The quantitative analysis of NRG was carried out by UV–Vis spectroscopy (FullTech Instruments, Rome, mod. PG T80), using 10.00 mm quartz cells (Hellma Italia S.r.l., Milano, Italia). The samples were opportunely diluted with ethanol and analyzed at λ_max_ of 289 nm.

The UV–Vis method was validated for linearity, sensitivity, and repeatability [72]. We prepared a stock solution of NRG in ethanol by solubilizing 10 mg in 50 mL of the solvent in a calibrated flask, obtaining a solution containing 200 μg/mL of NRG. Then, 5 mL of the stock solution was placed into a calibrated flask and diluted with 50 mL ethanol, obtaining 20 μg/mL NRG. This solution was scanned in the range of 200–600 nm to determine the maximum absorption.

A calibration curve was constructed in the concentration range of 28–0.93 μg/mL, starting from the stock solution, obtaining an R^2^ equal to 0.9952. Each concentration was prepared in six replicates and analyzed in triplicate. The precision of the analytical method was determined intra-day and inter-day by analyzing three of the calibration standards (28, 10, and 1 μg/mL) three times during the same day and the next three days. Limit of detection (LOD) and limit of quantitation (LOQ) were determined based on the Equations (4) and (5):

LOD = 3.3 σ/S = 0.017 μg/mL(4)

LOQ = 10 σ/S = 0.52 μg/mL(5)

where σ is the standard deviation of the intercept, and S is the slope of the calibration curve.

### 3.6. Physical Stability of NRG_10_-SLNs Formulation

The physical stability of the optimized NRG_10_-SLNs formulation was evaluated over time in different conditions of temperature and light. Briefly, the NRG_10_-SLNs suspension was maintained at 4° C or 25 °C in the dark and in daylight for 30 days. At timed intervals (1, 7, 15, and 30 days), the suspension was analyzed for mean particle sizes, PDI, and ζ.

### 3.7. Physical–Chemical Characterization of NRG_10_-SLNs

The TAQ500 equipment (TA Instruments, New Castle, DE, USA) was used to carry out thermogravimetric analysis (TGA) and differential scanning calorimetry analysis (DSC). TGA scans were performed using a 100 mL/min argon flow. Following a vacuum drying process, all samples were heated to 700 °C at a rate of 10 °C/min. DSC analysis was carried out at room temperature up to 350 °C at a heating rate of 5 °C/min, while nitrogen flow was 50 mL/min.

X-ray diffraction (XRD) experiments were run at room temperature using a Bragg–Brentano theta–2theta setup with Cu K_α_ radiation (40 V, 40 mA) on a Bruker D8 Advance diffractometer (Bruker, Karlsruhe, Germany). At a step of 0.2°/s, XRD patterns were obtained in the 5–80° range. A comparison was made between the diffraction peaks and the JCPDS (Joint Committee on Powdered Diffraction Standards) database.

The µ-Raman measurements were carried out on both NRG and NRG-SLNs systems, in the solid state using a portable spectrometer “BTR 111 Mini-RamTM” (B&W Tek, Inc., Newark, NJ, USA). This instrument operates with an excitation wavelength of 785 nm (laser diode), a maximum laser power of 280 mW, and a thermoelectrically cooled CCD detector. The laser output power has been appropriately adjusted to guarantee the best signal/noise ratio while maintaining low integration times. The µ-Raman spectra were acquired in the 65–3150 cm^−1^ range with a resolution of 10 cm^−1^ and an acquisition time of 10 s × 32 scans. To guarantee the best possible performance, a preliminary calibration procedure was carried out on the instrument, exploiting the peak at 520.6 cm^−1^ of a silicon chip. The spectrometer used is also equipped with a BAC151B Raman microscope on which an 80× objective was mounted, allowing microscopy measurements with a working distance of 1.25 mm and a laser spot size on the sample surface of ~25 µm. For our measurements, the maximum power delivered to the system was ~120 mW.

A Jeol JEM 2010F (JEOL, Ltd., Tokyo, Japan) transmission electron microscope (TEM) was used for the morphological characterization of lyophilized NRG_10_-SLNs, before and after redispersion. The experiments were performed at 200 kV of accelerating voltage.

### 3.8. Antibacterial Activity

The antibacterial activity experiments were performed with American Type Culture Collection (ATCC) reference strains. Specifically, *Staphylococcus aureus* ATCC 6538, methicillin-resistant *S. aureus* (MRSA) ATCC 43300, *Escherichia coli* ATCC 10536, and *Pseudomonas aeruginosa* ATCC 9027 were used as the representative Gram positive and Gram negative bacteria. The strains were stored at 70 °C in Microbanks™ (Pro-Lab Diagnostics, Neston, UK). Cultures were grown in Mueller–Hinton Broth (MHB, Oxoid) for 24 h before subjecting them to the assay.

The minimum inhibitory concentration (MIC) and the minimum bactericidal concentration (MBC) of free NRG, NRG_10_-SLNs, and empty SLNs were determined according to the guidelines issued by the Clinical and Laboratory Standards Institute, CLSI (2018) [75] with some modifications. The samples were diluted twofold in 96-well round-bottomed plates using MHB. Free NRG was assayed after solubilization in dimethylsulfoxide (DMSO). Overnight bacterial cultures were inoculated to yield a final concentration of 5 × 10^5^ CFU/mL, confirmed by viable counts. The MIC was considered as the lowest concentration of each sample to induce a complete inhibition of visible bacterial growth after incubation for 24 h. To evaluate the inhibition of metabolic bacterial activity, 20 μL of 2,3,5-triphenyl tetrazolium chloride (TTC) 0.125% (*w*/*v*) was added to all the wells, followed by 1 h of incubation. The tetrazolium salt is frequently employed in MIC determinations; when dissolved in water it is colorless, but it turns red when metabolically active bacteria are present. This red color is directly correlated with the number of living cells.

The MBC was determined by seeding 20 µL from all clear-colored MIC wells onto agar plates and was defined as the lowest concentration of compound that killed 99.9% of the inoculum. MHB and MHB with 1% DMSO were used as growth controls. As a positive drug reference standard, ofloxacin was employed. Modal results were computed by evaluating the data from a minimum of three replicates.

### 3.9. Effect on Biofilm Formation

The effect of NRG_10_-SLNs, free NRG, and empty SLNs on the biofilm-forming ability of *S. aureus* ATCC 6538 and *S. aureus* ATCC 43300 (MRSA) was tested on polystyrene flat-bottomed microtitre plates as previously described [76]. Overnight cultures in TSB + 1% glucose (TSBG) of *S. aureus* strains were adjusted in TSBG to 1 × 10^6^ CFU/mL and were dispensed into each well of 96-well polystyrene flat-bottomed microtitre plates containing twofold dilutions of the NRG_10_-SLNs, free NRG, and empty SLNs ranging from 0.5 × MIC to 0.125 x MIC. After incubation at 37 °C for 24 h, the planktonic phase was removed and each well was washed twice with sterile PBS (pH 7.4), dried, stained with 0.1% safranin, and washed with water. The stained biofilm biomass was resuspended in 30% (*v*/*v*) acetic acid and OD_492_ was measured using a spectrophotometer EIA reader. A biofilm control consisting of a TSBG medium was included. The reduction percentage of biofilm biomass was calculated using the following equation (Equation (6)):

100 − (mean OD_492_ of sample well/mean OD_492_ of control well) × 100 (6)

### 3.10. Statistical Analysis

Each value is expressed as mean ± S.D., and three separate tests were conducted for every analysis. A two-way analysis of variance (ANOVA) was used to analyze the data, and for multiple comparisons, a Bonferroni post hoc test was used [77]. A significant value was defined as *p* < 0.05.

## 4. Conclusions

In this work, SLNs were formulated to obtain a sustained release of NRG and an improvement in its antibacterial and antibiofilm effectiveness. NRG-SLNs formulations were prepared using solvent emulsification/diffusion and ultrasonication methods, selecting Compritol^®^ 888 ATO (COM) as the lipid and Tween 80 as the surfactant. The optimized NRG_10_-SLNs system exhibited excellent nanometer sizes (approximately 50 nm) and encapsulation efficiency (97.9%), due to the high affinity of NRG with the lipid used for the SLNs synthesis. TGA, DSC, XRD, and µ-Raman techniques confirm this strong interaction, responsible for the prolonged drug release in vitro. Finally, antibacterial activity studies were performed in vitro on Gram-positive and Gram-negative bacteria. An improvement in antibacterial activity for the NRG-loaded SLNs was observed against *S. aureus* strains, probably due to the interaction between the surfactant coating the SLNs and the bacterial surface. A similar trend was observed for the biofilm inhibition.

Our study demonstrates the efficacy of NRG_10_-SLNs as an innovative antibacterial system towards *S. aureus* strains, providing a basis for the development of a liquid form pharmaceutical dosage for the treatment of topical ocular infections. Ex vivo studies on excited rabbit cornea are in progress to confirm the potentiality of our system.

## Figures and Tables

**Table 1:** Encapsulation parameters (E.E. %, D.C. %) and Yield % of NRG-SLNs prepared with different theoretical amounts of NRG (namely, NRG_x_-SLNs, with x = 1, … 10%).

Sample	Yield % ± S.D.	E.E. % ± S.D.	D.C. % ± S.D.
Empty SLNs	48.1 ± 0.5	---	---
NRG_1_-SLNs	52.3 ± 0.4	89.2 ± 2.5	0.37 ± 0.05
NRG_2_-SLNs	54.8 ± 3.3	92.5 ± 0.3	0.73 ± 0.06
NRG_3_-SLNs	58.1 ± 7.8	95.6 ± 1.1	1.07 ± 0.16
NRG_4_-SLNs	46.8 ± 2.2	93.8 ± 2.0	1.72 ± 2.40
NRG_5_-SLNs	54.0 ± 3.1	94.6 ± 0.5	1.88 ± 3.25
NRG_6_-SLNs	52.5 ± 6.2	94.4 ± 2.1	2.65 ± 1.58
NRG_7_-SLNs	48.8 ± 4.2	92.5 ± 4.0	2.80 ± 0.80
NRG_8_-SLNs	48.6 ± 6.6	92.7 ± 0.5	3.26 ± 2.13
NRG_9_-SLNs	56.2 ± 5.2	94.2 ± 4.8	3.20 ± 1.20
NRG_10_-SLNs	51.5 ± 4.1	97.9 ± 0.7	4.11 ± 0.20

**Table 2:** Hydrodynamic diameters (D_H_), polydispersity index (PDI), and zeta potential (ζ) values in empty SLNs and NRG10-SLNs samples, before and after lyophilization.

Sample	D_H_ (nm) ± S.D.		PDI (%) ± S.D.		ζ (mV) ± S.D.	
	Before	After	Before	After	Before	After
Empty SLNs	54.4 ± 2.5	50.2 ± 4.2	0.12 ± 0.01	0.15 ± 0.02	−30.5 ± 0.6	−29.3 ± 1.4
NRG_10_-SLNs	58.8 ± 0.3	56.8 ± 6.2	0.15 ± 0.00	0.15 ± 0.00	−30.3 ± 0.1	−30.5 ± 2.6

**Table 3:** Hydrodynamic diameter (D_H_), polydispersity index (PDI), and zeta potential (ζ) values in the redispersed NRG_10_-SLNs formulation at different conditions of light and temperature.

Day	4° C			25° C in the Daylight			25 ° C in the Dark		
	D_H_ (nm) ± S.D.	PDI (%) ± S.D.	ζ (mV) ± S.D.	D_H_ (nm) ± S.D.	PDI (%) ± S.D.	ζ (mV) ± S.D.	D_H_ (nm) ± S.D.	PDI (%) ± S.D.	ζ (mV) ± S.D.
1	58.8 ± 0.3	0.15 ± 0.00	−30.3 ± 0.1	58.8 ± 0.3	0.15 ± 0.00	−30.3 ± 0.1	58.8 ± 1.3	0.15 ± 0.01	−30.3 ± 0.1
7	68.6 ± 3.1	0.15 ± 0.01	−28.1 ± 0.6	58.4 ± 3.6	0.13 ± 0.00	−31.2 ± 0.8	61.4 ± 0.6	0.14 ± 0.02	−29.2 ± 0.8
15	78.4 ± 1.6	0.12 ± 0.01	−27.8 ± 0.2	64.6 ± 0.5	0.12 ± 0.01	−29.9 ± 1.0	64.4 ± 6.4	0.14 ± 0.01	−28.6 ± 1.0
30	89.5 ± 9.3	0.14 ± 0.09	−24.5 ± 0.4	65.3 ± 3.7	0.15 ± 0.02	−28.9 ± 2.2	68.5 ± 2.8	0.15 ± 0.01	−27.9 ± 2.9

**Table 4:** Regression coefficient (R^2^), rate constant (K_0_, K_1,_ and K_H_ for zero-order, first-order, and Higuchi model, respectively), and transport exponent (n) of Korsmeyer-Peppas model.

Sample	Zero-Order		First-Order		Higuchi		Korsmeyer-Peppas	
	R^2^	K_0_ (d^−1^)	R^2^	K_1_ (d^−1^)	R^2^	K_H_ (d^−1/2^)	R^2^	n
NRG_10_-SLNs	0.8009	0.5665	0.9939	0.0275	0.9535	8.0534	0.72	0.5764

**Table 5:** Minimum inhibitory concentration (MIC) and minimum bactericidal concentration (MBC) of free NRG, NRG_10_-SLNs, and empty SLNs.

Strains	NRG_10_-SLNs		NRG		Empty SLNs	
	MIC	MBC	MIC	MBC	MIC	MBC
	mg/mL					
*S. aureus* ATCC 6538	0.5	>1	0.5	>1	1	>1
*S. aureus* (MRSA)ATCC 43300	0.25	>1	0.5	>1	0.5	>1
*E. coli* ATCC 10536	1	>1	1	>1	>1	>1
*P. aeruginosa* ATCC 9027	>1	>1	>1	>1	>1	>1

## Data Availability

We have no other data to share.

## Supplementary Materials

The following supporting information can be downloaded at: https://www.mdpi.com/article/10.3390/ph18020232/s1, Figure S1: Chemical structure of naringenin; Figure S2: Derivative thermogravimetric analysis.
