# Supplementary material for: Naringenin-Loaded Solid Lipid Nanoparticles: Physical–Chemical Characterization and In Vitro Antibacterial Activity
Source: Pharmaceuticals (Basel). 2025 Feb 8;18(2):232. doi: 10.3390/ph18020232 (PMC11859375; doi:10.3390/ph18020232)
Supplement: Supplementary file 1 [file pharmaceuticals-18-00232-s001.zip › pharmaceuticals-3390268-supplementary.pdf]

pharmaceuticals

Supplementary Materials

Naringenin-Loaded Solid Lipid Nanoparticles: Physical-Chemical Characterization and In Vitro Antibacterial Activity

Federica De Gaetano[1]*, Francesco Caridi[2], Noemi Totaro[1], Consuelo Celesti[3], Valentina Venuti[2], Giovanna Ginestra[1], Antonia Nostro[1], Silvana Tommasini[1], Cinzia Anna Ventura[1]*, Rosanna Stancanelli[1]

[1] Department of Chemical, Biological, Pharmaceutical and Environmental Sciences, University of Messina, V.le Ferdinando Stagno D'Alcontres 31, 98166 Messina, Italy; noemi.totaro@studenti.unime.it (N.T.); giovanna.ginestra@unime.it (G.G.); antonia.nostro@unime.it (A.N.); stommasini@unime.it (S.T.); rstancanelli@unime.it (R.S.)

[2] Department of Mathematical and Computer Sciences, Physical Sciences and Earth Sciences, University of Messina, V.le Ferdinando Stagno D'Alcontres 31, 98166 Messina, Italy; fcaridi@unime.it (F.C.); vvenuti@unime.it (V.V.)

[3] Department of Engineering, University of Messina, Contrada Di Dio, 98166 Messina, Italy; ccelesti@unime.it (C.C.)

* Correspondence: fedegaetano@unime.it (F.D.G); caventura@unime.it (C.A.V.)

Table of content

- **Figure S1.** Chemical structure of naringenin

- **Figure S2.** Derivative thermogravimetric analysis

Academic Editor: Kelong Fan

Received: 16 January 2025
Accepted: 6 February 2025
Published: 8 February 2025

Citation: De Gaetano, F.; Caridi, F.; Totaro, N.; Celesti, C.; Venuti, V.; Ginestra, G.; Nostro, A.; Tommasini, S.; Ventura, C.A.; Stancanelli, R. Naringenin-Loaded Solid Lipid Nanoparticles: Physical–Chemical Characterization and In Vitro Antibacterial Activity. *Pharmaceuticals* **2025**, *18*, 232. https://doi.org/10.3390/ph18020232

Copyright: © 2025 by the authors. Licensee MDPI, Basel, Switzerland. This article is an open access article distributed under the terms and conditions of the Creative Commons Attribution (CC BY) license (https://creativecommons.org/licenses/by/4.0/).

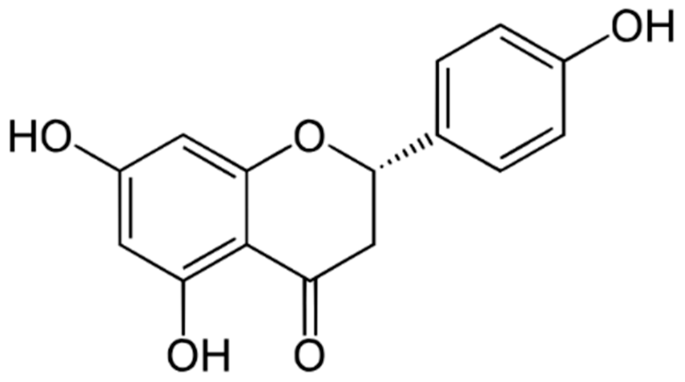

Figure S1. Chemical structure of naringenin

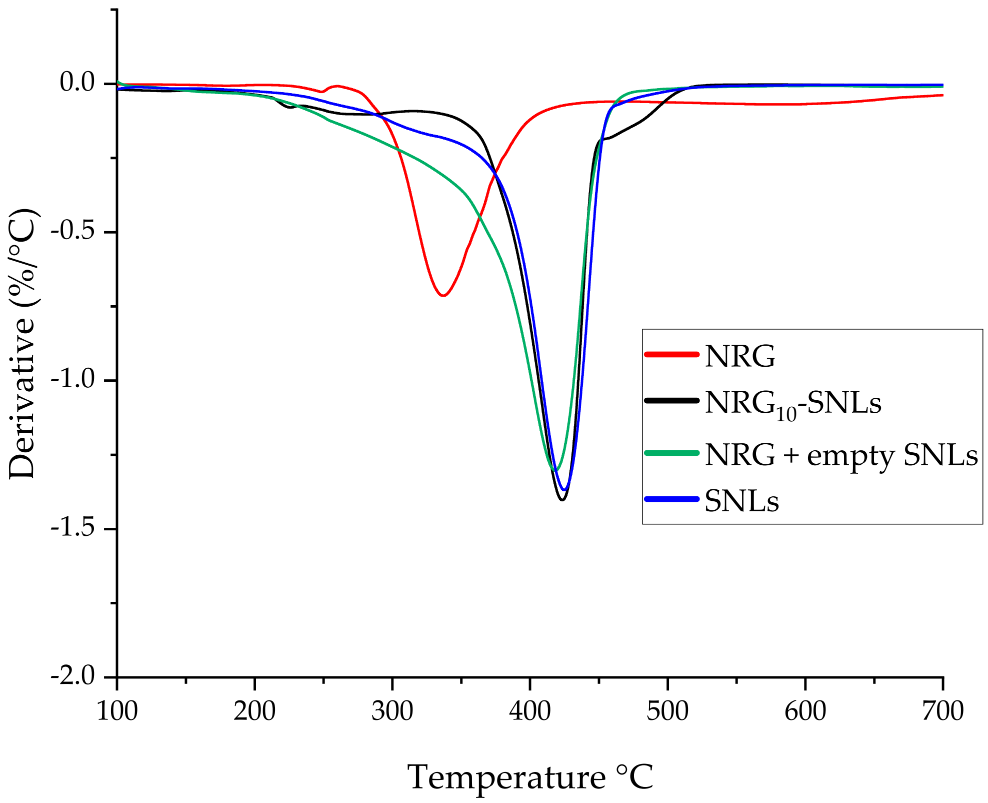

Figure S2. Derivative thermogravimetric analysis (DTG) of NRG (red line), NRG10-SLNs (black line), NRG+SLNs physical mixture (green line) and empty SLNs (blue line)

Disclaimer/Publisher's Note: The statements, opinions and data contained in all publications are solely those of the individual author(s) and contributor(s) and not of MDPI and/or the editor(s). MDPI and/or the editor(s) disclaim responsibility for any injury to people or property resulting from any ideas, methods, instructions or products referred to in the content.
